# Correlations of hematological parameters with bone marrow findings in chronic lymphoproliferative disorders associated with hepatitis viruses

**Published:** 2013-12-25

**Authors:** C Ciufu, V Arama, H Bumbea, C Dobrea, I Ion, AM Vladareanu

**Affiliations:** * Hematology Department, University Emergency Hospital, Bucharest; ** ”Matei Bals” Institute of Infectious Diseases, Bucharest; *** ”Victor Babes” National Institute of Development and Research, Bucharest; **** Neurology Department, University Emergency Hospital, Bucharest

**Keywords:** hepatitis viruses, chronic lymphoproliferative disorders, myelodysplasia, cytopenia

## Abstract

Abstract

Background. Hepatitis B and C viruses’ infections are often associated with hematological disorders in evolution, suggesting that these viruses have a tropism for peripheral blood and/or bone marrow cells.

Aim. To analyze the hematological parameters and bone marrow findings in a group of patients diagnosed with chronic lymphoproliferative disorders (CLD) and hepatitis viruses B, C, D infections, which were included in the research grant (acronym LIMFO-VIR) between December 2007 and May 2010 in the Hematology Department of the Emergency University Hospital of Bucharest.

Methods and results. Patients were diagnosed by using immunopathology according to the WHO criteria. The analyzed group included 42 patients (both sexes), with the mean age of 60,35 years. The most frequent hematologic disease was non-Hodgkin’s lymphoma 30/42 (71,42%), followed by chronic lymphocytic leukemia (16,66%) and Hodgkin’s lymphoma (7,14%). Hepatitis viruses were distributed: 17/42 (40,47%) patients with HBV, 22/42 (52,38%) with HCV and 3/42 (7,14%) had a double/triple association of viruses. Most of the patients had an indolent type of disease - 27/42 (64,28%), whereas 15/42 (35,71%) had an aggressive one, pattern found both in the HBV and HCV infected groups. An abnormal bone marrow result was revealed in 32/42 (76,19%) patients, 19 (59,37%) of them being HCV infected. Myelodysplasia was found in 6/42(14,28%) patients, the majority being HCV infected, all having an indolent form of CLD. The antiviral therapy did not influence the hematological parameters (no significant differences were found between the groups with/without an antiviral therapy).

Discussions. Patients with hepatitis virus infections may associate neutropenia and thrombocytopenia; the mechanisms are thought to involve hypersplenism, autoimmune processes and antiviral therapy. We excluded the influence of chemotherapy, as the study was performed before the treatment. In our group, patients whether HBV or HCV infected, presented an isolated cytopenia. The abnormal bone marrow cellularity (increased or decreased) and dysplasia were found especially in the HCV group. There are studies showing no association between myelodysplasia and hepatitis viruses; others found a strong relation of these. One of the mechanisms of myelodysplasia could be a dysregulation of the immune system.

Conclusions. Bone marrow/peripheral blood features correlate with the type of viral infection and HCV is more prone to develop additional hematological changes than HBV. The degree of bone marrow involvement by CLDs influences these features. We considered mandatory to perform a bone marrow analysis at the diagnosis of CLDs to stage and to establish if other bone marrow changes were present, a crucial aspect for therapy and outcome of the disease. The association between the hepatitis viruses – myelodysplasia- autoimmunity seems to have a role in the lymphoproliferative disorders etiology.

Abbreviations: CLD – chronic lymphoproliferative disorders; NHL- non-Hodgkin’s lymphoma, CLL- chronic lymphocytic leukemia, HL- Hodgkin’s lymphoma, MDS – myelodysplastic syndrome, AML – acute myeloid leukemia

## Background

Hepatitis B and C viruses infections represent an important public health problem, because of the increasing prevalence, evolution to chronic disease, cirrhosis and hepatocellular carcinoma, and also because of their association to autoimmune diseases and chronic lymphoproliferative disorders. The hematological changes can occur during hepatitis infections, such as aplastic anemia, granulocytopenia, thrombocytopenia or pancytopenia, thus suggesting an extrahepatic tropism for peripheral blood cells and bone marrow cells. The probable mechanism involves the viral replication within medullar progenitors, leading to cell differentiation and proliferation inhibition [**1**]. 

The association of chronic lymphoproliferative disorders with hepatitis viruses was analyzed in many epidemiological studies, in order to asses the hepatitis viruses involvement in the CLD pathogenesis. 

HBV has a hepatic tropism, but many studies revealed small quantities of non-replicative DNA-HBV in peripheral blood mononuclear cells (monocytes, B and T lymphocytes) and more seldom in polymorphonuclear cells [**2**-**6**]. These observations suggested that lymphocytes could represent an extrahepatic reservoir, the mechanism through which HBV genome replicates within lymphocytes is yet unknown [**6**,**7**]. Also, in chronic HBV infected patients, the DNA-HBV was identified in cultures performed on hematogenous bone marrow [**5**]. 

HCV is also capable of infecting and replicating in hematopoietic cells. Studies demonstrated RNA-HCV in T-lymphocytes, B-lymphocytes and monocytes of patients HCV infected [**1**,**8**]. HCV infection was associated with many extrahepatic diseases – type II/III mixed cryoglobulinemia, non-Hodgkin’s B-cell lymphomas. B-cell proliferation in HCV-infected patients is thought to be the result of chronic antigenic stimulation [**7**,**9**]. Another fact supporting the association of HCV infection and B-cell lymphoproliferative disorders is that anti viral therapy can lead to regression of splenic marginal zone lymphoma [**10**]. 

Patients with hepatitis virus infections develop abnormalities in peripheral cell counts, most commonly neutropenia and thrombocytopenia; the mechanisms are thought to involve hypersplenism, autoimmune processes [**11**] and antiviral therapy. The myelodysplastic syndrome may also occur in the evolution of hepatitis infections; recent data has shown that marrow failure in some cases of MDS is associated with autoimmunity; T-cell mediated myelosuppression and cytokine-induced cytopenias [**12**]. Part of the cytopenias associated with hepatitis infections could be secondary to myelodysplastic syndrome occurrence, due to ineffective hematopoiesis and immune processes. 

In this retrospective analysis of patients with chronic lymphoproliferative disorders and hepatitis virus infections, we followed the correlations of bone marrow findings with hematologic abnormalities and tried to see whether these could be explained partially by hepatitis viruses impact on bone marrow cells. 

## methods

The analyzed group was selected from patients of LIMFO-VIR grant, diagnosed with chronic lymphoproliferative disorders associating hepatitis viruses B, C, D, between December 2007 and May 2010 in the Hematology Department of the University Emergency Hospital of Bucharest [**13**]. All the patients had a positive diagnosis for CLD, established:

1. For chronic lymphocytic leukemia (CLL), according to the National Cancer Institute (NCI) - lymphocytosis > 5 x 109/L in peripheral blood and confirmation of the immunophenotype by flow-cytometry [**14**].

2. For non-Hodgkin’s lymphoma (NHL) – by assessing the immunophenotype of the malignant lymphoid population by immunohistochemistry, using modified REAL [**15**] and WHO classifications [**16**,**17**]. Lymph node and/or bone marrow biopsy were performed.

For hepatitis infections diagnosis, serological tests using ELISA method were positive.

Laboratory parameters. Complete blood count (CBC) was performed during the same period with the bone marrow analysis. Other biochemical and coagulation tests were performed - INR, ALT, total bilirubin and alkaline phosphatase. Also, abdominal ultrasound was performed to all patients; in order to asses the organomegaly. 

Bone marrow analysis. In our group, 36 patients had bone marrow biopsy and 6 patients had bone marrow aspirate. The bone marrow biopsies were evaluated in “V. Babes” National Institute of Research and Development, by a specialized pathologist. In cases with BM aspirate, a complete cellular differential was performed - 500 cells – in Hematology Laboratory of Hematology Department of the University Emergency Hospital Bucharest. Each case was evaluated for cellularity, clonal lymphocytosis, plasmacytosis, dysplasia.

Statistical analysis. The software used for statistic analysis was SPSS 19 and Excel 2007. Statistical significance was established using Student t-test for numeric clinical values. Fisher’s exact test was used for categorical variables. 

## Results

In the analyzed group of 42 patients, 25 were female (59,50%) and 17 male (40,50%), with a mean age of 60,35 years (range: 30-84) at the hematological diagnosis. The bone marrow biopsy was performed in order to establish the diagnosis and/or to stage the disease. The hematologic diseases distribution was: 3/42 (7,14%) patients with classic Hodgkin’s lymphoma - one with mixed cellularity, one with lymphocyte depletion and one with lymphocyte predominance, 2/42 (4,76%) patients with Waldenstrom macroglobulinemia, 7/42 (16,66%) – chronic lymphocytic leukemia, 30/42 (71,42%) patients having non-Hodgkin’s lymphoma – 28 (93,33%) patients with B-NHL and 2 (6,66%) patients with T-NHL. In B-cell NHL group, we found the following histological types: 12 marginal type, 9 diffuse large cell, 1 diffuse small cell, 2 mantle cell, 2 follicular, and 2 lymphocytic type.

Hepatitis viruses were distributed within our group: 17/42 (40,47%) patients with HBV, 22/42 (52,38%) patients with HCV and 3/42 (7,14%) patients had double/triple association of viruses.

**Fig. 1:**
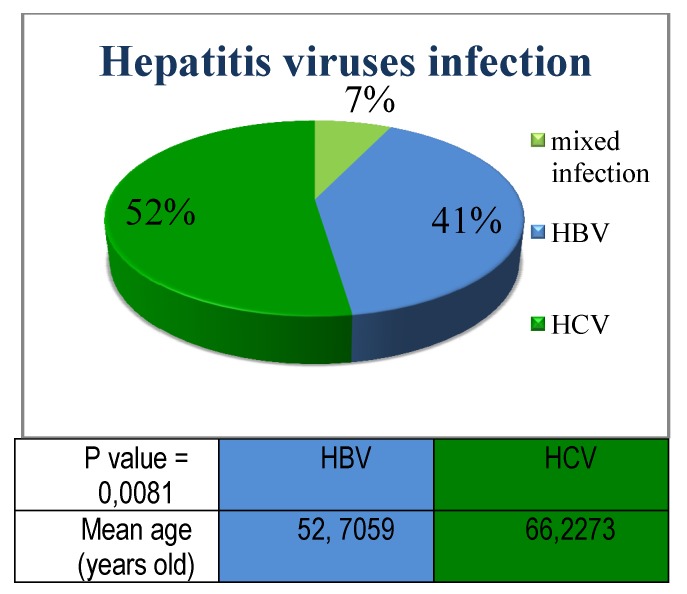
Hepatitis viruses’ distribution: HCV infection had a higher frequency compared to HBV infection; the mean age of patients diagnosed with HCV infection was significantly increased compared to the HBV group

The HCV infection was found in 16 patients with NHL, 3 with CLL, 1 with HL and 2 with Waldenstrom macroglobulinemia.

**Fig. 2:**
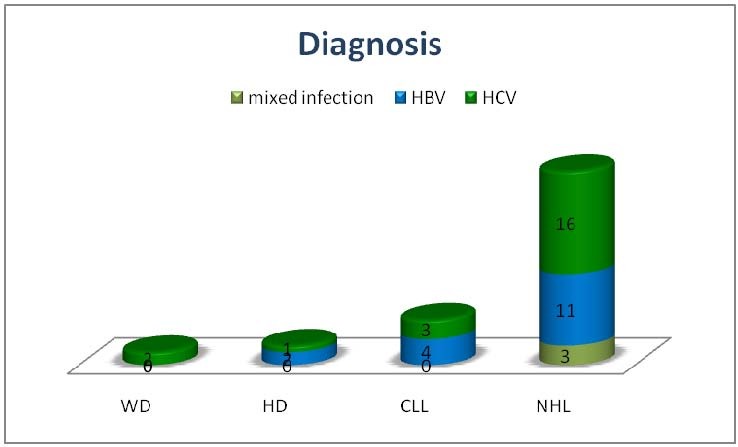
Distribution of CLDs according to the viral infection: non-Hodgkin’s lymphoma was predominant in the studied group, mainly associated with the HCV infection

According to the histological type of the hematological disease, 15/42 (35,71%) had an aggressive type and 27/42 (64,28%) had an indolent form of the disease. Out of 17 patients with HBV – 7 (41,17%) have an aggressive hematological disease and 10 (58,8%) – an indolent form; out of 22 patients with HCV – 7 (31,81%) patients had an aggressive disease and 15 (68,18%) an indolent disease. Out of 3 patients with association of hepatitis viruses – 2 patients have an indolent form and 1 patient – an aggressive form of the disease. 

**Fig. 3:**
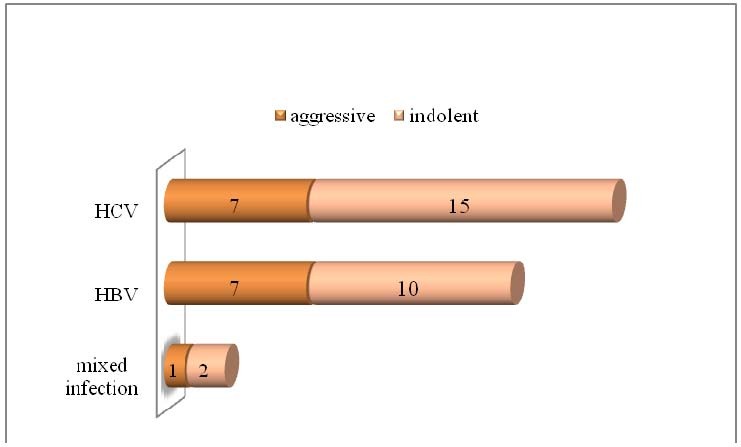
The association between the histological type of CLD and hepatitis viruses: in all cases of infection with hepatitis viruses B, C or mixed infection, the indolent form of the hematological disease was more frequent

The viral infection was documented before the hematological disease in 16/42 (38,09%) patients. 18/42 (42,85%) patients were diagnosed with CLD and hepatitis viral infection at the same time or within 3 month, whereas 8/42 (19,04%) patients were diagnosed in more than 3 months after the hematological diagnosis. Within the group with documented previous infection, HCV was more frequent – 7 patients with indolent CLD and 2 patients with aggressive disease, compared to HBV – 3 patients with indolent disease and 2 patients with aggressive CLD. Also, HVC was more found in the group of concomitant diagnosis of CLD and hepatitis infection (6 HCV infected patients versus 2 HBV infected patients, associating indolent type of CLD). The latter group is very likely to include patients having undiagnosed previous viral infection at the time of CLD diagnosis, the viral infection being detected because patients were screened for it. If we count together both groups, it means that the majority of patients have a history of hepatitis infection. HBV was predominant in the group diagnosed in more than 3 months after the hematological diagnosis (5 patients – all with indolent CLD, compared to 3 HCV infected patients – 2 indolent and 1 aggressive CLD). 

**Hematological and biochemical parameters**

CBC was available for all patients in our study; we analyzed the occurrence of cytopenias. According to the World Health Organization (WHO), cytopenias are defined as hemoglobin value under 10 g/dl, neutrophil count under 1,8 x 109/l and platelet count under 100 x 109/l. Thus, 12/42 (28,57%) patients had anemia and 2 patients had severe anemia (hemoglobin values under 7 g/dl, with a minimum of 4,7 g/dl). Only 9/42 (21,42%) patients were thrombocytopenic and two of these had severe thrombocytopenia, under 10.000/mmc. Four out of 42 patients (9,52%) presented with leukopenia (values under 4000/mmc), whereas 21 patients (50%) had an increased number of leukocytes (values over 8000/mmc). Neutrophil count under 1800/mmc was revealed in 7/42 (16,66%) patients. Isolated cytopenia was found in 11/42 (26,19%) patients – 4 with HVB infection, 6 with HCV infection and 1 patient with double infection. 5 patients (11,90%) had bicytopenia – 2 HBV infected, 2 HCV infected and one with association of hepatitis viruses. Only one patient (2,38%) had pancytopenia (and HBV infection). 

**Fig. 4:**
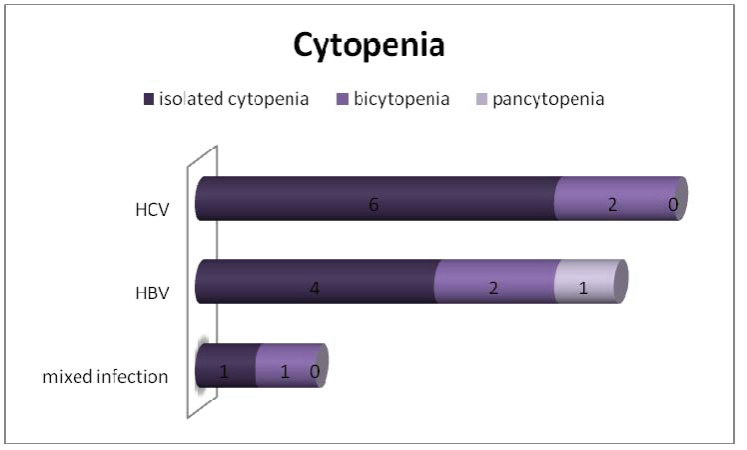
The association of cytopenias and hepatitis viruses: isolated cytopenia was the most frequent CBC abnormality, associated mainly to HCV infection; pancytopenia was found in only one patient which had HBV infection

We also evaluated the hepatic function. Therefore, alkaline phosphatase was increased in 8/42 (19%) patients, total bilirubin over limit in 18/42 (42,85%) patients, ALT also increased in 18 patients; 26/42 (61,90%) patients had an increased INR.

Hepatomegaly and splenomegaly were assessed by abdominal ultrasound; 31/42 (73,80%) patients had splenomegaly and 34/42 (80,95%) had hepatomegaly.

**Table 1:**
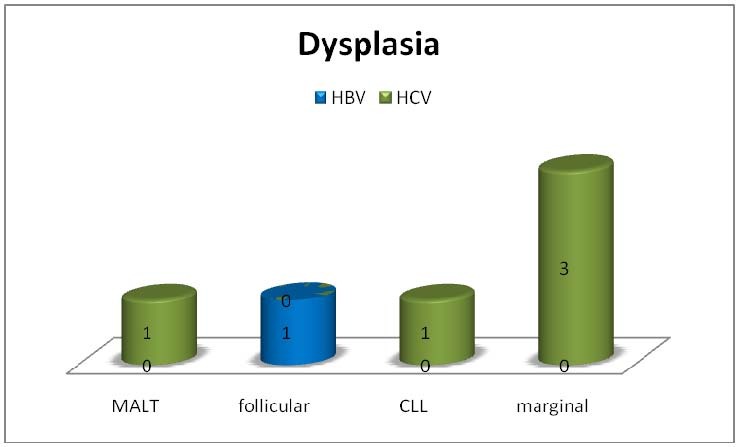
Clinical and laboratory characteristics (mean values)

**Bone marrow findings**

An abnormal bone marrow was revealed in 32/42 (76,19%) patients.

24/42 (57,14%) patients had an increased bone marrow cellularity, 2 patients had a hypocellular bone marrow and 16/42 (38,09%) – normal BM cellularity. Only one patient had erythroid hyperplasia (over 35% nucleated red blood cells precursors in bone marrow, whereas 14/42 (33,33%) patients had a decreased erythroid lineage. Megakaryocytic hyperplasia was revealed in 2 patients (4,76%) and a decreased megakaryocytic lineage was found in 12 patients (28,57%). Clonal lymphocytosis was present in the majority of patients – 28/42 (66,7%), ranges 10-85%. Myelofibrosis, evaluated only on bone marrow biopsy, was found in only one patient.

The following signs of dysplasia were evaluated: dyserythropoiesis– nuclear irregularities, nuclear budding, internuclear bridging; dysmyelopoiesis – abnormal lobulation and granulation, and/or dysmegakaryopoiesis - hypolobulation, multinucleated forms; myelodysplasia was positively diagnosed when over 10% cells on either lineage presented the described signs. Dysplasia was found in 6 out of 42 patients (14,3%); five patients presented dysmyelopoiesis (hypogranullarity, left shift) and one patient had dysmegakaryopoiesis (hypolobulation). HCV infection was present in 5 of the patients with dysplasia and HBV in one patient. All of the 6 dysplasia patients had a non-aggressive histological type of CLD (3 marginal, 1 MALT, 1 follicular, 1 CLL) and all of them have had the viral infection diagnosed before the CLD diagnosis (time range 1-17 years). 

**Fig. 5:**
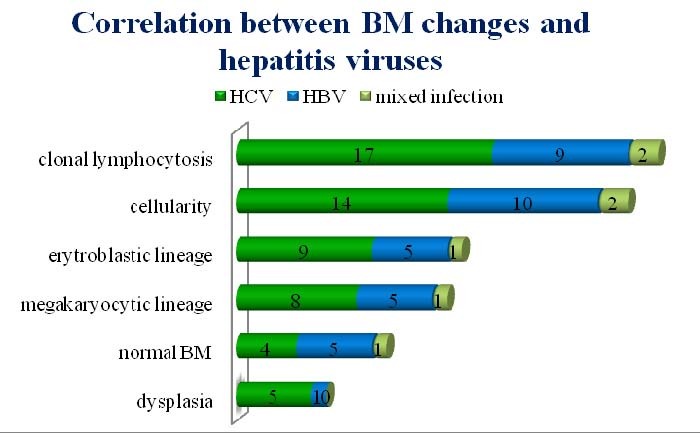
The presence of the bone marrow dysplasia in patients with lymphoproliferative condition and hepatitis viruses: although signs of dysplasia were found in only few patients, almost all (5 of 6 patients) had HCV infection; all the cases presented an indolent form of CLD

 Analyzing the distribution of hepatitis viruses in the group of patients with bone marrow abnormalities, we observed that HCV was involved in 17 cases of clonal lymphocytosis, compared to HBV which was found in only 9 cases and mixed infection in only 2 cases. Modified cellularity (increased or decreased) was found in 14 cases of HCV infection and 10 cases of HBV infection; changes in erythroid and megakaryocytic lineage were detected in 9 (erythroid) and 8 (megakaryocytic) cases of HCV infection, and respectively with equal distribution for HBV (5 cases for each). As we previously showed, dysplasia is associated with 5 cases of HCV infection and only one case of HBV. Our results suggest that HCV is more frequently associated with bone marrow changes in patients with chronic lymphoproliferative disorder.

**Fig. 6:**
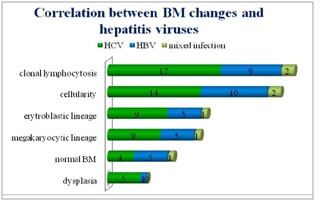
Bone marrow abnormalities and hepatitis viruses: HCV is more frequently found in patients with bone marrow changes compared to HVB

Within the group of patients with abnormal bone marrow, 19/32 (59,4%) patients are HCV positive. Of them, 16 patients were diagnosed with hepatitis virus infection before or concomitant to CLD diagnosis, time ranges from 1 year to 17 years.

**Correlations between laboratory and bone marrow findings**

From patients with isolated cytopenia, none had dysplasia and most of them had reduced erythroid and megakaryocytic lineage and also clonal lymphocytosis. Only one patient with marginal NHL and HCV infection presented bicytopenia (anemia and thrombocytopenia), associating also dysplastic bone marrow. We found one patient with pancytopenia, in whom bone marrow was hypercellular through clonal lymphocytosis. This patient had HBV infection and mantle cell NHL. 

**Table 2:**
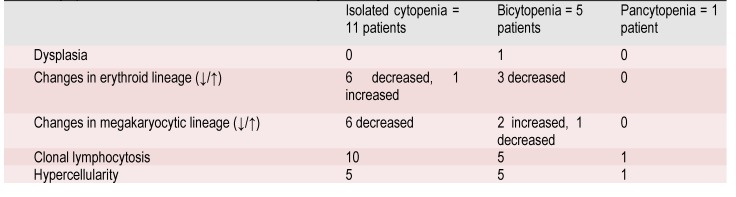
Cytopenia’s association with bone marrow findings

Regarding the peripheral counts’ abnormalities, a strong correlation was found between HBV infection and neutropenia /leucopenia (OR=9/OR=3,7), while HCV was linked to clonal lymphocytosis (OR=2,4).

**Table 3:**
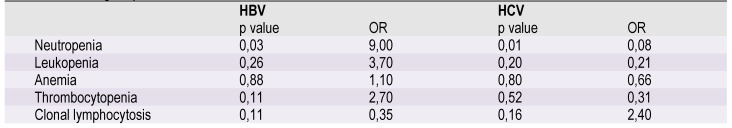
Hematological parameters correlated to viral infection

Antiviral therapy was given in 15/42 (35,71%) patients – 10 patients with HBV infection received Lamivudine/Entecavir, the rest of the patients – 2 with mixed viral infection and 3 patients with HCV infection - received combined antiviral therapy. No significant differences were found in peripheral cell counts of patients with or without antiviral therapy.

**Table 4:**
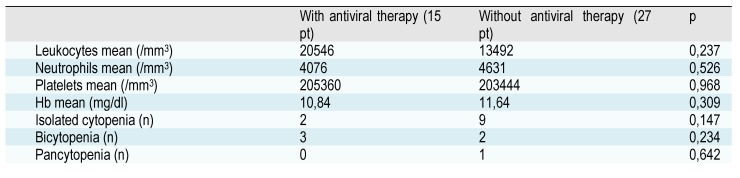
Correlations of hematological parameters and antiviral therapy

## Discussion

It is well known that hematological disorders may occur during hepatitis viral infections, such as aplastic anemia, granulocytopenia, thrombocytopenia or even pancytopenia, thus suggesting an extrahepatic tropism of the viruses for peripheral blood cells and bone marrow cells. The most likely mechanism involves viral replication within medullar progenitors, leading to cell differentiation and inhibition of proliferation [1].

 The relationship between CLDs and hepatitis viruses infections has been established by many epidemiological studies, the majority of these conducted in countries where the prevalence of hepatitis infection is high [**18**,19], but also studies from countries with lower prevalence found a positive association between CLD and hepatitis viruses [**20**].

Here we report an analysis of hematological parameters and bone marrow findings in patients with CLDs associated with hepatitis viruses infections (B,C,D) at the onset of hematological disease. Therefore, neither hematological parameters, nor bone marrow features are modified by specific therapy for the hematological disease, as the analysis was performed on data collected before chemotherapy was given. This suggests that all changes in peripheral counts and bone marrow are due to the hematological disease, the virus infection and associated antiviral therapy (if present), and not to additional chemotherapy, which is otherwise known to influence the hematological features. 

In our group of patients, the HCV infection was more frequent (22 from 42 patients); cytopenias presented only 8 patients HCV infected – 6 patients with isolated cytopenia and 2 patients with bicytopenia. We also found 7 HBV infected patients with peripheral cytopenias: 4 patients - isolated cytopenia, 2 patients - bicytopenia and 1 patient with pancytopenia. Two patients with double viral infection had cytopenias (1 isolated and 1 bicytopenia). As we already know, patients with hepatitis virus infections may have neutropenia and thrombocytopenia; the cause of thrombocytopenia is multifactorial in many patients: hypersplenism, reduced thrombopoiesis as a result of decreased production of endogenous thrombopoietin by the liver, marrow suppression by hepatitis viruses, dysregulation of immunity [**21**]. Drug-induced cytopenias may occur (secondary to antiviral therapy).

HBV was correlated to neutropenia, but no particular evidence of a link between HBV and neutropenia in the literatures we found.

From 32 patients presenting changes on bone marrow analysis, 19 have HCV infection. The clonal lymphocytosis was associated in 17 patients HCV-infected, comparing to only 9 HBV infected patients and 2 mixed viral infection patients; it was interpreted as being involvement of lymphoproliferative disorder. The majority of patients has been diagnosed with hepatitis infection before/concomitant to CLD, with time range 1-17 years. Considering the epidemiological studies as an argument favoring hepatitis viruses having a role in CLD pathogenesis, we can presume that at least a part of our patients have viral infection as a risk factor and one of etiological agents of hematologic disease. Regarding the histological type of CLD, the indolent form was more frequent in our group. Pellicelli et al revealed that the indolent HCV-related B-NHL is associated with a longer exposure to HCV infection and DLBCL - with a shorter duration of HCV exposure [**22**]. Our results are in agreement with the literature data, the indolent type of CLD being more frequent in patients with long history of hepatitis infection. Regarding the patients diagnosed with hepatitis infection in more than 3 months after CLD diagnosis, it is possible that the viral screening was performed late (meaning not at the time, nor within 3 months after CLD diagnosis) during the evolution of the hematologic disorder, since it was not a routine many years before, therefore these patients could be wrongly categorized as not having a hepatitis infection at the time of CLD diagnosis. Interestingly, HBV was more frequent than HCV within the group diagnosed with hepatitis infection in more than 3 months after the CLD diagnosis. A possible explanation is the lack of the complete HBV screening routine (HBs antigen, HBs antibodies, HBc antibodies), leading to “escape” of occult /resolved/serological “window” HBV infection diagnosis, these patients being diagnosed with HBV infection only at the time of the viral reactivation (AgHBs sero-reversion); it means that the number of patients with a previous hepatitis infection can be higher than expected, this having epidemiological and possible ethiopathogenic implications. 

Dysplasia was found in 5 patients with HCV infection and one patient with HBV infection (14,28%), all with indolent form of chronic lymphoproliferation.

 A retrospective Egyptian study on the epidemiology of myelodysplastic syndrome found 13% patients positive for HCV, considering this fact an association rather than a true relation, because of high prevalence of HCV among Egyptians [**23**]. Murashige et al conducted a hospital based case-control study to evaluate the association between myeloid malignancies and hepatitis viruses; they found no association and that HCV is unlikely to increase the risk for myeloid malignancies, including MDS [**24**]. Still, this study had a few “problems”, one of them being the small number of HCV positive patients, limiting the interpretation of the study results. Confuting these data, an U.S. study on over 61.000 patients with hematologic malignancies – data collected from The Surveillance, Epidemiology and End Result SEER –Medicare – found HCV to be associated with 1,5 -fold increased risk for acute myeloid leukemia and myelodysplastic syndromes, and HBV was not linked by any lymphoid or myeloid malignancy [**25**]. The Korean large case-control study over the association of hepatitis viruses and hematologic malignancies found HBV having an important role in the pathogenesis of both lymphoid and myeloid malignancies, more than HCV; this fact could be explained by the high prevalence of HBV in Korea, compared to HCV prevalence (1%) [**19**]. Also, about 5% of the population is infected prenatally (meaning a longer duration of HBV carriage), compared to the US population who is infected during adulthood, and also have a lower prevalence of hepatitis infections. The different results of the above mentioned studies are explained by these. Another large case-control Swedish study proved an increased risk for AML and MDS in patients with prior history of infections, including HCV infection, and autoimmune conditions; also, the infections and autoimmune disorders were found independent risk factors for AML/MDS [**26**]. These novel data bring the etiopathogenesis of MDS into another light. Any abnormality of immune system, which plays an important role in tumor surveillance, will allow tumor cells to evade immune response and progress into malignancy; the cytokines produces by inflammatory cells have a pro-tumor activity and produce genomic instability. Even if autoimmune phenomena follow the diagnosis of MDS in general, the autoimmunity should be considered a possible causative factor of myeloid malignancies, not only a complicating factor.

The relationship between autoimmune disorders and the development of CLDs have been already established by studies and involve dysregulation of the immune system, triggering the transformation of normal polyclonal lymphocytes into monoclonal population, leading to lymphoproliferative disorders.

 A special feature of patients with MDS is the high incidence of malignant tumors, including lymphoma, than the general population [**27**,**28**] reflecting an underlying defect in immune surveillance, that led to the emergence of MDS clone in the first place [**29**].

Whether the coexistence of MDS clone and lymphoproliferative disorder in our patients is related or not, we do not know. It is possible that MDS precede and favor the occurrence of CLD (because of immune abnormalities) or they could have no particular relation, just coexisting like two independent clones. There are a few reports in the literature about concomitant diagnosis of lymphoid and myeloid malignancies [**30**-**32**] and future studies should establish if there is any link between their pathogenesis and risk factors underlying the diseases.

An interesting fact is that all the patients having dysplasia and CLD had a hepatitis infection diagnosed before CLD diagnosis, time range 1-17 years. This raises the question if the longer exposure to virus infection could also favor myelodysplasia, not only CLD occurrence. 

## Conclusions

In our group, HCV was more frequent than HBV, possibly involved in B-cell lymphoproliferative disorder etiology (considering the 21 patients with previous HCV infection and longer exposure), but also in other changes such as bone marrow dysplasia and peripheral cytopenias.

Considering that antiviral therapy does not influence the hematological parameters (no significant differences were found between the groups with/without antiviral therapy), we can establish that bone marrow /peripheral counts features strictly depend on the type of viral infection – HCV is more prone to develop additional hematological changes compared to HBV, and the degree of bone marrow involvement by chronic lymphoproliferative condition. It is mandatory to perform a bone marrow analysis at diagnosis of chronic lymphoproliferative disorder, not only for staging the disease, but also in order to establish if additional changes (myelodysplasia) other than medullar clonal lymphocytosis are present, as an important element for future therapy (many lymphoma therapeutic agents being involved in myelodysplasia occurrence), and evolution of the disease. The association between hepatitis viruses – myelodysplasia- autoimmunity seems to have a role in lymphoproliferative disorders etiology, but further studies are needed to strongly confirm this hypothesis.

**Disclosures**. None.
